# Closing the diagnostic gap in medical mycology: The LODDY Test for identification of *Lodderomyces elongisporus*

**DOI:** 10.1093/mmy/myaf076

**Published:** 2025-08-22

**Authors:** Watcharamat Muangkaew, Natthapaninee Thanomsridetchai, Panyawat Boontanom, Pornpan Khum-eam, Marut Tangwattanachuleeporn, Sumate Ampawong, Passanesh Sukphopetch

**Affiliations:** Department of Microbiology and Immunology, Faculty of Tropical Medicine, Mahidol University, Bangkok 10400, Thailand; Department of Medical Technology, Faculty of Allied Health Sciences, Burapha University, Chon Buri 20131, Thailand; Educational Technology and Art Unit, Office of Dean, Faculty of Tropical Medicine, Mahidol University, Bangkok 10400, Thailand; Department of Microbiology and Immunology, Faculty of Tropical Medicine, Mahidol University, Bangkok 10400, Thailand; Department of Medical Science, Faculty of Allied Health Sciences, Burapha University, Chon Buri 20131, Thailand; Department of Tropical Pathology, Faculty of Tropical Medicine, Mahidol University, Bangkok 10400, Thailand; Department of Microbiology and Immunology, Faculty of Tropical Medicine, Mahidol University, Bangkok 10400, Thailand

**Keywords:** *Lodderomyces elongisporus*, fungal bloodstream infections, differential medium, LODDY Test, fungal diagnostics, *Candida* species misidentification

## Abstract

*Lodderomyces elongisporus* is an emerging, cryptic opportunistic yeast increasingly linked to fungemia, especially in immunocompromised patients and those with indwelling devices. Frequently misidentified as *Candida parapsilosis* due to phenotypic overlap, it escapes timely diagnosis, delaying effective therapy. Despite accounting for <2% of candidemia cases, true prevalence is likely underestimated. Critically, *L. elongisporus* exhibits reduced susceptibility to echinocandins, making misidentification clinically dangerous. To develop and validate the LODDY Test—a novel, cost-effective, pH-indicator-based culture medium for rapid, accurate identification of *L. elongisporus*, particularly in resource-limited settings. The LODDY Test integrates four differential carbohydrates (sucrose, maltose, lactose, and arabinose) with bromocresol purple. We tested 41 *L. elongisporus* isolates (1 reference, 40 internal transcribed spacer (ITS) region -confirmed environmental strains) and 40 comparator yeasts (*C. parapsilosis* sensu stricto, *C. albicans, C. tropicalis*, and *Nakaseomyces glabratus*) from urban/peri-urban Thai sites. Species identity was confirmed by ITS sequencing. LODDY results were benchmarked against the Analytical Profile Index 20C Auxanographic assimilation test (API 20C AUX), CHROMagar™ chromogenic medium, and Matrix-Assisted Laser Desorption/Ionization Time-of-Flight Mass Spectrometry (MALDI-TOF MS), and ITS-DNA sequencing. The LODDY Test achieved 100% sensitivity and specificity, distinctly identifying *L. elongisporus* (no color change) from yellow-producing *Candida* spp. and purple *N. glabratus*. Performance matched MALDI-TOF MS and ITS sequencing but required only basic equipment. The LODDY Test is a low-cost, high-accuracy diagnostic tool suitable for decentralized labs. Its clinical validation in bloodstream isolates is the next step toward improving candidemia outcomes in low-resource settings.

## Introduction

### Fungal bloodstream infections and the diagnostic crisis

Fungal bloodstream infections (BSIs) represent a mounting global health threat, particularly among hospitalized patients, individuals receiving immunosuppressive therapies, and those with indwelling medical devices.^1–5^ The incidence of opportunistic fungal infections has steadily increased over the past two decades, driven in part by expanded use of invasive medical procedures, prolonged intensive care unit (ICU) stays, immunosuppressive regimens, and the growing population of patients with underlying comorbidities, including malignancies and organ transplants. Among these infections, candidemia remains the most prevalent, accounting for the majority of fungal BSIs worldwide.^6–8^

### *Lodderomyces elongisporus* as an emerging pathogen

While *Candida albicans* has received the most clinical attention, non-albicans *Candida* species and other emerging yeasts now present significant diagnostic challenges.^9–12^*Lodderomyces elongisporus*, formerly considered a non-pathogenic environmental organism, has emerged as a clinically significant pathogen causing life-threatening bloodstream infections. It shares considerable phenotypic and biochemical similarity with *C. parapsilosis*, a common cause of catheter-associated fungemia, leading to frequent misidentification and underreporting in clinical laboratories.^9,12–16^

Misidentification is not a benign error. *Lodderomyces elongisporus* exhibits partial resistance to echinocandins, while *Candida parapsilosis* also displays higher Minimum Inhibitory Concentration (MICs) than *C. albicans*. Overlooking these pharmacologic differences can result in suboptimal therapy, treatment delays, and increased morbidity. Invasive fungal infections caused by emerging yeasts are associated with mortality rates exceeding 40% when appropriate therapy is delayed.^17–19^ Thus, early and accurate identification of *L. elongisporus* is critical for optimizing outcomes and guiding antifungal stewardship. Although fluconazole resistance is well documented in *C. parapsilosis*, most *L. elongisporus* isolates remain susceptible. However, occasional bloodstream isolates have shown reduced susceptibility, underscoring the risk of resistance development under antifungal pressure, particularly in immunocompromised patients.^9,12–19^ In this study, comparator strains were limited to *C. parapsilosis* sensu stricto; *C. orthopsilosis* and *C. metapsilosis* were excluded to minimize phylogenetic variability and to focus on the species most often misidentified as *L. elongisporus* in clinical practice.^9,12–19^

### Current diagnostic limitations in identifying *L. elongisporus*

Traditional fungal identification relies mainly on phenotypic and biochemical testing—such as colony morphology on Sabouraud glucose agar,^9,12^ chromogenic media,^9,17^ and sugar assimilation profiles like API 20C AUX.^9^ While inexpensive and widely available, these methods lack discriminatory power for closely related yeasts. In our study, CHROMagar™ *Candida* failed to reliably differentiate *L. elongisporus*, although reports suggest CHROMagar™ BBL *Candida* may yield distinctive blue colonies. A comparative evaluation of this medium with the LODDY Test across diverse laboratory settings could better inform diagnostic strategy.^9,12,17,20^

Biochemical assays, though semi-automated and convenient, also fail to distinguish *L. elongisporus* from *C. parapsilosis* due to overlapping sugar assimilation profiles. Their accuracy is further undermined by variability in growth conditions, incubation times, and inter-laboratory procedures, leading to frequent misclassification. Such errors affect not only individual patient care but also broader epidemiological surveillance.^9,12^

Advanced approaches such as MALDI-TOF MS and ITS-DNA sequencing deliver high-resolution identification.^9,14,17,20^ However, their high cost, need for sophisticated equipment, and reliance on trained personnel limit accessibility, especially in urgent or resource-limited contexts. Even in well-equipped laboratories, batch processing and turnaround time can constrain clinical utility.

In many low- and middle-income countries (LMICs), where fungal infection burdens are highest but resources are most constrained, diagnostic workflows still depend heavily on morphology- and biochemistry-based methods. As a result, misidentification remains common, delaying treatment, worsening outcomes, and undermining antifungal stewardship. Bridging this diagnostic gap is therefore a pressing challenge in global health and medical mycology.

### The clinical and epidemiological relevance of *L. elongisporus*

Epidemiological data on the prevalence of *L. elongisporus* remain limited despite increasing clinical awareness. In a recent multi-center review, it represented <2% of yeast bloodstream isolates but was often misidentified as *C. parapsilosis* in automated biochemical systems.^9,12^ This indicates that current surveillance likely underestimates its true burden. Retrospective molecular reclassification has shown that many isolates once identified as *C. parapsilosis* were, in fact, *L. elongisporus*. Although surveillance reports suggest rarity, the species is more likely underdiagnosed than uncommon. Its recovery from soil, air, avian excreta, catheter tips, and bloodstream samples demonstrates both environmental ubiquity and nosocomial potential.^12,20–22^

The clinical significance of *L. elongisporus* is underscored by case reports documenting fungemia, endocarditis, and deep-seated infections.^23–26^ Unlike *C. albicans*, which readily forms hyphae, *L. elongisporus* shows limited filamentation—a trait that may facilitate immune evasion and colonization of prosthetic surfaces.^9,12,27–30^ Partial resistance to echinocandins further complicates empirical treatment, especially when species-level identification is lacking. Elevated echinocandin MICs, similar to those of *C. parapsilosis*, heighten the risk of misdirected therapy. Although *in vitro* MICs are elevated, some clinical efficacy remains plausible; nonetheless, misidentification can delay targeted therapy or trigger unnecessary escalation.^17–19^

This diagnostic blind spot has major implications for patient outcomes and public health. In laboratories unable to distinguish cryptic yeasts, delayed or inappropriate therapy contributes to higher morbidity and mortality. There is an urgent need for accurate, accessible, and affordable diagnostic approaches capable of detecting *L. elongisporus* and other emerging pathogens in real time.

### Study objectives and scientific significance

This study aimed to validate the diagnostic performance of the LODDY Test using a taxonomically and ecologically diverse panel of yeast isolates. The evaluation included 41 *L. elongisporus* strains (both reference and environmental), alongside clinical isolates of *C. albicans, C. tropicalis, C. parapsilosis* sensu stricto, and *Nakaseomyces glabratus*. The primary objective was to assess the test's clinical utility in terms of sensitivity, specificity, and reproducibility. To establish scientific credibility, results were benchmarked against conventional and advanced identification platforms—API 20C AUX, CHROMagar™, MALDI-TOF MS, and ITS region sequencing. By addressing key diagnostic gaps, this study supports the integration of the LODDY Test into routine workflows, enhancing streamlined identification and reducing misclassification risks in fungal diagnostics.

### Study design and scope

This study employed a structured, multi-phase experimental design to evaluate the diagnostic performance, reproducibility, and translational relevance of the LODDY Test in differentiating *L. elongisporus*. Rather than restating the study's objectives, this section focuses on the rationale for isolate selection, testing sequence, and alignment with molecular confirmation methods. A panel of clinical and environmental yeast isolates was assembled and systematically assessed through phenotypic assays, chromogenic hue response profiling, and ITS-based molecular identification. All experiments were conducted under controlled laboratory conditions to ensure consistency. Reproducibility was evaluated by independent observers across multiple replicates. As all specimens were obtained from routine diagnostic procedures and fully anonymized, institutional ethics approval was not required.

### Microorganisms and culture conditions

A total of 81 yeast isolates were included in this study, comprising 41 *L. elongisporus* isolates and 40 non-*L. elongisporus* isolates commonly implicated in misidentification. The *L. elongisporus* group consisted of the reference strain ATCC 11503 and 40 environmental isolates collected from bird droppings, air, and soil, all of which were confirmed by ITS-DNA sequencing. The non-*L. elongisporus* group included *C. albicans* (*n* = 10), *C. tropicalis* (*n* = 10), *C. parapsilosis* sensu stricto (*n* = 10), and *N. glabratus* (formerly *C. glabrata*) (*n* = 10). All isolates were subcultured on SDA and incubated at 37°C for 48 h. Following incubation, colonies were suspended in sterile 0.85% NaCl solution and adjusted to a 2 McFarland standard (∼10^7^ CFU/ml) for use in subsequent experiments.

### Development and optimization of the LODDY Test

The LODDY Test was developed as a pH-indicator-based differential medium tailored to exploit the unique carbohydrate metabolism of *L. elongisporus*, particularly its inability to ferment arabinose. The medium incorporated four discriminative carbohydrates—sucrose, maltose, lactose, and arabinose—at 10 g/l each, with bromocresol purple (0.4 g/l) serving as a pH-sensitive colorimetric indicator. The base matrix consisted of meat peptone (10 g/l), yeast extract (2 g/l), and agar (20 g/l).

To preserve the indicator's functionality, agar was autoclaved separately and combined aseptically with the carbohydrate mixture—adjusted to pH 6.0 ± 0.3—after cooling to 50°C. This formulation enabled species-specific chromogenic responses, facilitating expedited and visually interpretable identification of *L. elongisporus*. Prepared LODDY plates were stored at 4°C and used within 4 weeks. All inoculated plates were incubated at 30°C for 48 h, unless otherwise stated, to allow optimal chromogenic differentiation.

### Inoculation and incubation

Each yeast isolate was tested using two inoculation methods to evaluate both colony morphology and metabolic activity. First, standard streaking was performed using a sterile loop to observe colony morphology and associated color changes. Second, drop inoculation was carried out by spot-inoculating 10 µl of yeast suspension onto the LODDY Test medium to directly assess carbohydrate metabolism and resulting pH shifts. Yeast suspensions were standardized to 1.0 × 10^6^ CFU/ml by adjusting turbidity to a 0.5 McFarland standard, and 10 µl of each was spot-inoculated onto the surface of the LODDY medium. All inoculated plates were incubated aerobically at 37°C for 48 h. Following incubation, colony growth, colorimetric responses, and morphological characteristics were systematically documented for analysis.

### Interpretation of the LODDY Test results

Interpretation of the LODDY Test results was based on visible pH-induced color changes surrounding the colonies. *Lodderomyces elongisporus* exhibited no color change, corresponding to a neutral pH, while *C. albicans, C. tropicalis*, and *C. parapsilosis sensu stricto* produced a yellow coloration indicative of acidic metabolic byproducts. In contrast, *N. glabratus* generated a distinct purple hue, reflecting an alkaline pH profile. High-resolution photographs of the cultured plates were captured under standardized lighting conditions to ensure reproducibility and facilitate inter-laboratory comparison.

In addition to qualitative visual inspection, hue values were quantitatively assessed using image analysis software (Fiji Is Just ImageJ; version 2.14.0; National Institutes of Health, Bethesda, MD, USA). Photographs were taken with a DSLR camera using fixed settings (white balance, exposure, and distance) under standardized LED lighting. For each isolate, ten independent LODDY Test plates were analyzed. The region of interest (ROI) encompassing the colony and its surrounding medium was selected, and the average hue values were extracted using the HSB (Hue, Saturation, and Brightness) color model. Data were expressed as mean hue ± standard deviation. This semi-quantitative approach not only corroborated the visual interpretations but also enhanced reproducibility and provided a platform for future integration with digital or AI-assisted diagnostic tools.

### Colony-centered RGB and Hue quantification

To enhance precision and minimize color distortion from the peripheral agar background, we performed colony-centered image analysis based on a defined ROI. Specifically, the central 50% of each culture plate image—encompassing the densest colony growth and its immediate periphery—was extracted for color profiling. This center-focused ROI better reflects human visual focus and color perception under diagnostic conditions.

Digital images were analyzed using Fiji (Fiji Is Just ImageJ; version 2.14.0; National Institutes of Health, Bethesda, MD, USA). Mean Red, Green, and Blue (RGB) values were calculated from the cropped ROI, and mean RGB values were calculated from the cropped ROI. RGB values were normalized to the uninoculated media-alone control to quantify the chromatic shifts attributable to fungal metabolic activity. Subsequently, RGB values were converted to hue values (°) using the HSB color model to yield a single, interpretable metric of color tone.

To ensure consistency, all images were acquired using identical camera settings, including aperture, white balance, exposure, and fixed focal length, under controlled LED illumination (color temperature: 5600 K). Ten replicates per fungal species were processed independently.

Normalization against the media control allowed for ΔHue and ΔRGB computation, enhancing the interpretability of subtle color differences. This approach also facilitates future automation through computer vision pipelines or integration with explainable AI platforms for fungal diagnostics.

### Comparative evaluation with conventional methods

The diagnostic performance of the LODDY Test was compared with four standard yeast identification methods: API 20C AUX, CHROMagar™ *Candida*, MALDI-TOF MS, and ITS-DNA sequencing.

For biochemical identification using API 20C AUX, yeast suspensions adjusted to 2 McFarland were inoculated into API strips, incubated at 30°C for 48–72 h, and interpreted using the manufacturer's database software. For phenotypic differentiation, isolates were streaked onto CHROMagar™ *Candida* and incubated at 37°C for 48 h, with colony morphology and color assessed against reference standards.

Proteomic identification via MALDI-TOF MS involved protein extraction using a standardized ethanol/formic acid/acetonitrile protocol, followed by analysis with the Bruker Biotyper® system (version 3.1; Bruker Daltonics, Billerica, MA, USA), where identification scores ≥ 2.0 were considered species-level matches. All 41 isolates were confidently identified as *L. elongisporus*.

For molecular confirmation, genomic DNA was extracted using the Qiagen DNA Mini Kit, and the ITS1–5.8S–ITS2 region was amplified using ITS1 and ITS4 primers. PCR products were subjected to Sanger sequencing and aligned against GenBank and MycoBank databases for definitive species identification. ITS-DNA sequencing was performed on two randomly selected isolates, and the resulting sequences have been deposited in GenBank (Accession numbers PV855759 and PV855760).

All methods—LODDY Test, API 20C AUX, CHROMagar™, MALDI-TOF MS, and ITS sequencing—yielded concordant species-level identifications as *L. elongisporus*, validating the robustness of the diagnostic framework used in this study.

### Statistical analysis

Statistical analyses were performed using GraphPad Prism version 9. Diagnostic accuracy of the LODDY Test was assessed through the calculation of sensitivity, specificity, positive predictive value (PPV), and negative predictive value (NPV). Agreement between the LODDY Test and the molecular gold standard (ITS-DNA sequencing) was evaluated using Cohen's Kappa coefficient. To compare diagnostic performance across different identification methods, one-way analysis of variance (ANOVA) was conducted, followed by Tukey's post hoc test for pairwise comparisons. A *P* < .05 was considered statistically significant.

### Ethical considerations

This study was reviewed and approved by the Institutional Biosafety Committee (IBC) of Burapha University, Thailand (Approval No. IBC 013/2564). All experimental procedures involving microbial handling were conducted in accordance with established biosafety regulations and institutional guidelines to ensure laboratory safety and compliance with ethical standards.

## Results

### Performance of the LODDY Test in identifying *Lodderomyces elongisporus*

**Figure 1.**
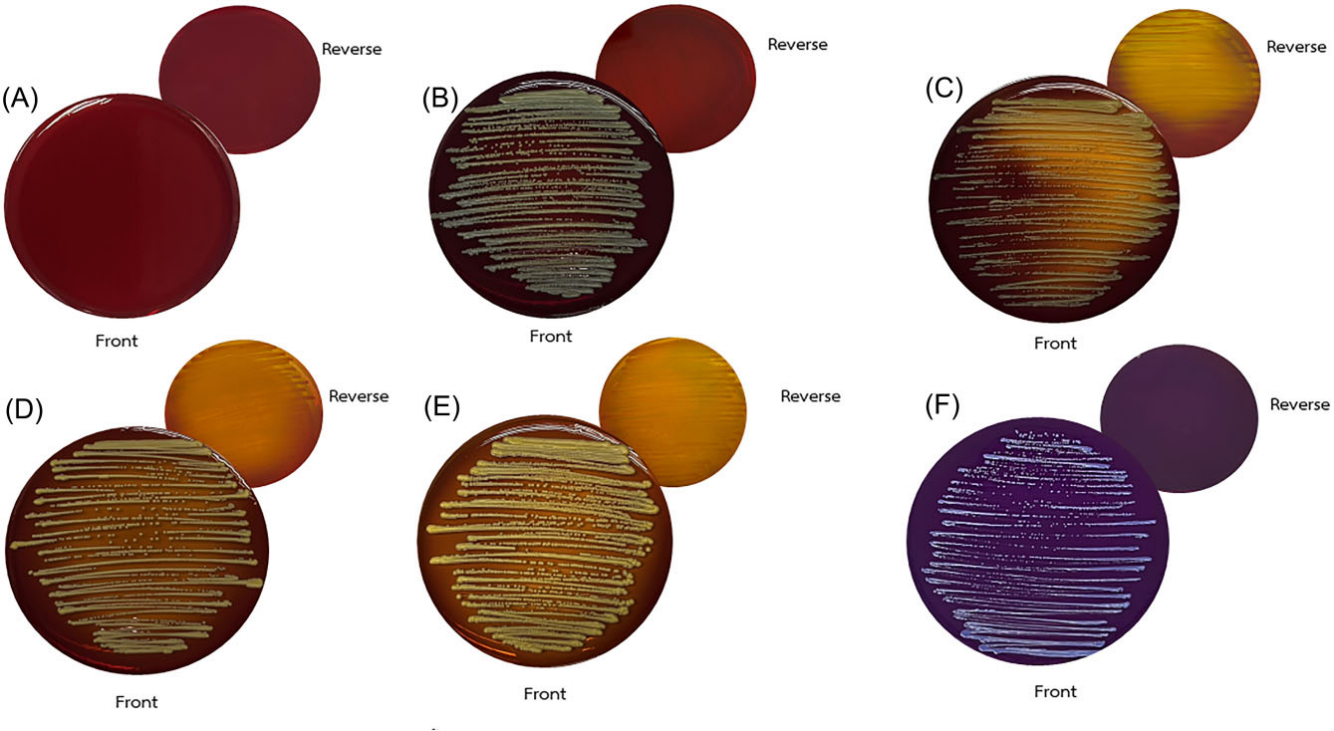
Distinctive chromogenic signatures of clinically significant yeasts cultured on LODDY medium. Representative colony morphologies of five medically important yeasts grown on LODDY Test medium after 48 h of incubation at 37°C. (A) Uninoculated medium exhibited no detectable chromogenic shift, confirming its neutral pH baseline. (B) Lodderomyces elongisporus ATCC 11503 similarly retained a neutral profile, indicating minimal acidification. In contrast, (C) Candida parapsilosis, (D) C. albicans, and (E) C. tropicalis triggered strong acidification, evident as low-pH pigmentation changes. (F) Nakaseomyces glabratus showed an opposite trend, producing a high-pH associated pigmentation shift consistent with alkalinization. These species-specific chromogenic patterns, quantified in Table 1 through RGB and hue values, underscore the diagnostic utility of the LODDY medium in practical yeast differentiation..

The LODDY Test reliably distinguished *L. elongisporus* from phylogenetically and phenotypically similar *Candida* species by leveraging distinct, species-specific pH-induced colony color changes. Following 48 h of incubation at 37°C, all 41 *L. elongisporus* isolates—including the ATCC 11503 reference strain—consistently exhibited no visible color change, reflecting a neutral pH profile associated with the organism's inability to ferment arabinose (Figure 1, Table 1).

**Table 1.** Colony-focused RGB and Hue analysis of yeast isolates on LODDY medium.

Sample	Mean R	Mean G	Mean B	Mean Hue (°)	ΔR	ΔG	ΔB	ΔHue (°)
Media alone	71.0	27.74	34.24	351.0	0.0	0.0	0.0	0.0
*Lodderomyces elongisporus*	52.61	19.16	14.4	7.65	−18.39	−8.59	−19.84	−343.35
*Candida parapsilosis*	110.02	64.35	24.96	25.3	39.02	36.61	−9.28	−325.7
*Candida albicans*	101.92	59.47	15.29	30.55	30.92	31.73	−18.96	−320.45
*Candida tropicalis*	134.88	83.09	25.39	31.51	63.89	55.35	−8.85	−319.49
*Nakaseomyces glabratus*	36.56	15.83	33.6	308.47	−34.44	−11.91	−0.64	−42.53

In contrast, non-*L. elongisporus* species exhibited distinct chromogenic responses that aligned with their respective metabolic profiles. *Candida albicans, C. tropicalis*, and *C. parapsilosis* produced a bright yellow coloration, reflecting the accumulation of acidic byproducts from active carbohydrate fermentation. In comparison, *Nakaseomyces glabratus* (formerly *C. glabrata*) generated a deep purple hue, indicative of an alkaline metabolic output likely associated with limited carbohydrate utilization.

All photographs were captured under standardized lighting to ensure visual consistency and inter-laboratory comparability. These pH-driven chromogenic signatures provide a practical, cost-effective, and visually interpretable tool for species-level differentiation, especially suited to low-resource diagnostic settings.

### Colony-centered Hue analysis validates visual discrimination

To enhance objectivity and align results with human visual perception, colony-focused hue quantification was performed using the HSB color model. Only the central region of each plate—encompassing the colony and its immediate surroundings—was analyzed. The media-alone control exhibited a baseline hue of approximately 351°, corresponding to a reddish-purple (Sangria) spectrum.

*Lodderomyces elongisporus* yielded a mean hue of 7.65°, which was nearly indistinguishable from the control (ΔHue = –343.35°), confirming the chromogenic neutrality observed by eye. In contrast, *C. tropicalis* and *C. parapsilosis* produced hue values of 31.51° and 25.30°, respectively, indicating orange-yellow shifts consistent with acidic metabolic byproducts. *C. albicans* demonstrated a hue of 30.55°, closely aligning with *C. parapsilosis* and reinforcing its similar metabolic output. Notably, *N. glabratus* exhibited a hue of 308.47°, representing a deep violet-blue tone and marking a substantial chromatic departure from the control baseline. These findings substantiate that colony-centered hue measurement more faithfully mirrors visually perceived color differences than whole-plate averaging, particularly in distinguishing species with pigmented or high-contrast growth.

### Objectivity and reproducibility in chromogenic interpretation

To further validate the diagnostic reliability of the LODDY Test, average hue values were measured from ten independent culture plates per species using standardized imaging software (ImageJ). The hue measurements consistently mirrored visual assessments. *Lodderomyces elongisporus* maintained near-neutral hues, with mean values ranging approximately from 275° to 360°. In contrast, *Candida* species demonstrated yellow-associated hues averaging between 25° and 32°, consistent with their acidogenic metabolic profiles. *N. glabratus* exhibited hue values near 308°, aligning with the violet spectrum and reflecting a distinct chromogenic output.

This reproducible colorimetric pattern confirms the LODDY Test as a robust, semi-quantitative platform for the streamlined identification of *L. elongisporus*, and highlights its future potential for integration with AI-assisted digital diagnostics.

These findings reaffirm the LODDY Test as a timely, highly specific, and cost-effective diagnostic platform for distinguishing *L. elongisporus* from other clinically significant yeast pathogens. Several smartphone applications—such as Color Grab™ (Loomatix) for Android and Color Name AR™ for iOS—can already extract HSB values from captured images in real time. These tools could be adapted to read LODDY Test plates, enabling hue-based fungal identification even in resource-limited settings. Integration with a standardized hue reference library could further support semi-automated, app-based diagnostics.

### Comparative analysis of LODDY Test with conventional diagnostic methods

To assess diagnostic performance, the LODDY Test was directly compared with four widely used yeast identification methods: API 20C AUX, CHROMagar™ *Candida*, MALDI-TOF MS, and ITS-DNA sequencing. While the molecular methods (MALDI-TOF MS and ITS sequencing) served as reference standards, the LODDY Test achieved equivalent diagnostic accuracy and outperformed conventional biochemical and chromogenic systems in both sensitivity and specificity (Table 2).

**Table 2.** Comparative diagnostic performance and feasibility of five yeast identification methods.

Method	Sensitivity (%)	Specificity (%)	PPV (%)	NPV (%)	Correct Identification	Misidentified as *Candida parapsilosis*	Turnaround time	Equipment required	Cost per sample	LMIC suitability
LODDY Test	100	100	100	100	41 (100%)	0 (0%)	48 h	Basic microbiology setup	Minimal cost/Basic-level infrastructure	Yes
API 20C AUX	84.2	89.5	88.9	85.7	34 (84.2%)	7 (15.8%)	72 h	Incubator, API reader	Moderate cost/ Mid-level infrastructure	Yes
CHROMagar™ *Candida*	78.9	85.3	81.4	82.1	32 (78.9%)	9 (21.1%)	48–72 h	Incubator		Yes
MALDI-TOF MS	100	100	100	100	41 (100%)	0 (0%)	<1 h	Mass spectrometer	High cost/Advanced instrumentation required	No
ITS-DNA Sequencing	100	100	100	100	41 (100%)	0 (0%)	48–72 h	PCR machine, DNA sequencer		No

This table summarizes the diagnostic accuracy, misidentification rates, turnaround time, cost, and equipment requirements of five yeast identification platforms. The LODDY Test achieved perfect diagnostic performance (100% sensitivity, specificity, positive predictive value [PPV], and negative predictive value [NPV]) without misclassifying any *Lodderomyces elongisporus* isolates. It outperformed conventional assays (API 20C AUX, CHROMagar™) and demonstrated greater feasibility than molecular tools (MALDI-TOF MS, ITS sequencing) in resource-limited settings. (PPV, positive predictive value; NPV, negative predictive value; LMICs, low- and middle-income countries.)

### Sensitivity, specificity, and diagnostic accuracy

The LODDY Test demonstrated 100% sensitivity and specificity in identifying *L. elongisporus*, with complete concordance with ITS-DNA sequencing, the molecular gold standard (Table 2).

The LODDY Test outperformed both API 20C AUX and CHROMagar™ *Candida*, which showed reduced specificity and higher misidentification rates, largely due to phenotypic similarities between *L. elongisporus* and *C. parapsilosis*. In contrast, the LODDY Test delivers comparable diagnostic precision with significantly lower operational requirements and cost, making it a more feasible option for broad implementation.

### Misidentification rates in API 20C AUX and CHROMagar™

Among the 41 *L. elongisporus* isolates tested, misidentification occurred in 15.8% of cases using API 20C AUX and in 21.1% of cases using CHROMagar™ *Candida*. The majority of misidentified isolates were incorrectly classified as *C. parapsilosis*, likely due to overlapping metabolic and phenotypic characteristics (Table 2).

These findings underscore the limitations of conventional biochemical and chromogenic media in reliably distinguishing *L. elongisporus* from phenotypically similar Candida species.

### Comparison of turnaround time and cost-effectiveness

One of the key advantages of the LODDY Test lies in its optimal balance between diagnostic efficiency, affordability, and operational simplicity (Table 2). Among available methods, MALDI-TOF MS offers the fastest turnaround time—typically under 1 h—but its high capital cost, maintenance requirements, and need for trained personnel limit its feasibility in low-resource settings. ITS-DNA sequencing, considered the molecular gold standard, provides definitive species identification; however, its turnaround time of 48–72 h and substantial per-test cost render it unsuitable for routine use in most clinical laboratories.

In contrast, while the 48-h processing time of the LODDY Test does not rival the speed of molecular platforms, it nonetheless constitutes a meaningful improvement over traditional biochemical methods, which often exceed 72 h. Importantly, this time frame remains clinically actionable, particularly in the context of bloodstream infections. When deployed as part of an early yeast screening workflow, the LODDY Test facilitates early, species-directed antifungal therapy—enhancing patient care and stewardship outcomes in settings where advanced molecular tools are inaccessible.

### Reproducibility and inter-laboratory validation

To evaluate reproducibility and robustness, the LODDY Test was independently validated in three microbiology laboratories across separate institutions. All 41 *L. elongisporus* isolates—including environmental and reference strains—were correctly identified in each laboratory, yielding 100% inter-laboratory concordance. No false-positive or false-negative results were reported, confirming the test's high reproducibility under varied laboratory conditions (Table 2).

### Statistical analysis and agreement with molecular methods

Agreement between the LODDY Test and ITS-DNA sequencing (the molecular gold standard) was evaluated using Cohen's Kappa coefficient, which yielded a value of κ = 1.00, indicating perfect agreement.

To compare diagnostic performance across methods, one-way ANOVA demonstrated a statistically significant difference in misidentification rates between the LODDY Test, API 20C AUX, and CHROMagar™ (*P* < .001). Tukey's post hoc analysis further confirmed that the LODDY Test performed significantly better than conventional biochemical methods (*P* < .001), reinforcing its superior diagnostic accuracy (Table 2).

To contextualize the diagnostic relevance of the LODDY Test within current clinical practice, we provide a consolidated comparison of five widely used yeast identification methods in Table 2. This summary integrates not only diagnostic performance indicators—such as sensitivity, specificity, and misidentification patterns—but also operational metrics critical to real-world implementation, including turnaround time, equipment requirements (notably incubator use for culture-based platforms), cost per test, and applicability in LMIC settings. The inclusion of such parameters reflects the practical constraints frequently encountered in decentralized laboratories, where access to molecular infrastructure remains limited.

Given the LODDY Test's demonstrated species-level specificity, visually interpretable readout within 48 h, and alignment with molecular confirmation, this comparative framework reinforces its unique position among existing tools. Unlike conventional biochemical assays that frequently misidentify *L. elongisporus* as *C. parapsilosis*, and molecular methods that—while accurate—are often resource-prohibitive, the LODDY Test offers an intermediate solution that balances diagnostic fidelity with accessibility. As shown in Table 2, this balance of precision, cost-efficiency, and ease of implementation makes the LODDY Test particularly well-suited to resource-constrained environments where early antifungal intervention and fungal surveillance are most urgently needed.

## Discussion

*Lodderomyces elongisporus* has long been dangerously mischaracterized in global diagnostics. The LODDY Test breaks this silence by introducing the first pH-indicator-based medium specifically tailored to this cryptic pathogen, long obscured by phenotypic overlap and misidentified as *Candida parapsilosis*.^9,12,32^ Unlike conventional tools, it achieves species-level precision without molecular instrumentation, addressing a critical diagnostic gap in under-resourced settings.^32^

In head-to-head comparisons, the LODDY Test matched molecular gold standards while remaining low-cost and accessible (Table 2). Exploiting arabinose non-utilization, it generated distinctive chromogenic signatures: yellow for *C. albicans, C. tropicalis*, and *C. parapsilosis sensu stricto*; purple for *Nakaseomyces glabratus*; and neutral for *L. elongisporus*. These results were reproducible across laboratories and fully concordant with ITS sequencing, underscoring reliability and robustness.^32^

Unlike biochemical assays and chromogenic agars that frequently misidentify *L. elongisporus*,^9,12,32^ the LODDY Test eliminates diagnostic ambiguity. While MALDI-TOF MS and ITS sequencing remain definitive,^12,35^ their costs, turnaround times, and infrastructure needs limit routine use. By offering molecular-level precision without resource barriers, the LODDY Test represents not just an alternative but a breakthrough in the fight against fungal sepsis.

Our findings confirm that the LODDY Test achieves 100% sensitivity and specificity across 41 isolates, outperforming conventional media that often misclassify *L. elongisporus* as *C. parapsilosis*. The addition of hue quantification enhances reproducibility and opens the door to smartphone- or AI-assisted platforms, enabling future point-of-care diagnostics even in decentralized laboratories.

Beyond its primary role, the LODDY Test revealed distinct chromogenic responses among other medically important yeasts, suggesting potential for broader diagnostic application. Such reproducible colorimetric patterns could be particularly valuable in laboratories lacking molecular tools. Moreover, the assay strengthens global fungal surveillance by enabling early detection of underreported species, advancing antifungal stewardship, and promoting diagnostic equity.^9,12,32–35^

Future validation should include related species within the *C. parapsilosis* complex (*C. orthopsilosis* and *C. metapsilosis*) and clinical bloodstream isolates to confirm translational applicability. Minor inter-observer variability may occur, but integration with smartphone-based or AI-assisted imaging can standardize interpretation and expand accessibility. Incorporating the LODDY Test into LMIC diagnostic workflows alongside microscopy and antifungal susceptibility testing would strengthen laboratory capacity without requiring specialized infrastructure.

In summary, Loddy redefines fungal diagnostics by achieving molecular-level precision through a simple, affordable, and scalable platform. It does not merely fill a gap; it reshapes the diagnostic landscape. More than a laboratory tool, this innovation represents a transformative step toward equitable and accessible fungal healthcare worldwide.

In conclusion, the LODDY Test redefines the role of culture-based diagnostics in contemporary medical mycology by delivering molecular-level accuracy through a simple, affordable, and scalable platform. By bridging a critical diagnostic gap in the identification of *L. elongisporus*, this novel tool enables earlier and more accurate treatment, strengthens antifungal stewardship, and enhances diagnostic equity—especially in resource-limited settings where such tools are most urgently needed.

As emerging fungal pathogens continue to challenge existing diagnostic systems, innovations like the LODDY Test exemplify how targeted, cost-conscious diagnostics can yield high clinical and public health impact. Future studies incorporating bloodstream isolates, clinical outcome data, and digital integration will be pivotal in further establishing the LODDY Test as a frontline diagnostic assay for invasive yeast infections worldwide.

### Limitations

Despite the promising performance of the LODDY Test, several limitations warrant acknowledgment. First, this study primarily utilized environmental isolates and the ATCC reference strain, without testing clinical bloodstream isolates from patients with documented fungemia. Consequently, future validation studies incorporating clinical isolates are essential to confirm diagnostic accuracy, robustness, and practical utility in real-world clinical settings.

Second, although interpretation of colorimetric changes is generally straightforward, potential factors such as environmental contamination, mixed cultures, or weak metabolic responses could occasionally lead to misinterpretation. Additionally, observer variability in color perception may arise, particularly under suboptimal lighting conditions or due to individual perceptual differences. To mitigate these discrepancies, standardized training protocols, visual reference charts, or integration of smartphone-based digital imaging coupled with artificial intelligence-driven color analysis are recommended. These enhancements could further refine diagnostic consistency, minimize subjective interpretation bias, and substantially improve diagnostic accuracy.

Finally, while *C. parapsilosis sensu stricto* was included in the current validation, genetically related species such as *C. orthopsilosis* and *C. metapsilosis*—which share close phylogenetic and phenotypic characteristics—were not tested. Inclusion of these cryptic species in future studies will be essential to confirm the test's discriminatory power and exclude the possibility of cross-reactivity within the *C. parapsilosis* complex.

## Declaration of interest

The authors have no relevant financial or non-financial interests to disclose.

## Data Availability

The datasets generated and analyzed during the current study are available from the corresponding author on reasonable request. Raw data are not publicly shared due to institutional data protection policies and ethical restrictions concerning the use of clinical and environmental isolates. However, aggregated data supporting the findings of this study are available from the corresponding author upon reasonable request. The ITS sequences generated in this study have been deposited in GenBank under accession numbers PV855759 and PV855760.
